# Developing a diagnosis calculator to estimate the probability of bacterial pneumonia

**DOI:** 10.1017/ash.2023.408

**Published:** 2023-08-07

**Authors:** Jonathan D. Baghdadi, Ravi Tripathi, Lisa Pineles, Anthony D. Harris, Danica Palacio, Drew Charles, Kimberly C. Claeys, Emily Heil, Jackie Bork, Daniel J. Morgan

**Affiliations:** 1 Department of Epidemiology and Public Health, University of Maryland School of Medicine, Baltimore, MD, USA; 2 Division of Infectious Diseases, Department of Medicine, University of Maryland School of Medicine, Baltimore, MD, USA; 3 VA Maryland Healthcare System, Baltimore, MD, USA; 4 Department of Pharmacy Practice and Science, University of Maryland School of Pharmacy, Baltimore, MD, USA

## Abstract

Misdiagnosis of bacterial pneumonia increases risk of exposure to inappropriate antibiotics and adverse events. We developed a diagnosis calculator (https://calculator.testingwisely.com) to inform clinical diagnosis of community-acquired bacterial pneumonia using objective indicators, including incidence of disease, risk factors, and sensitivity and specificity of diagnostic tests, that were identified through literature review.

## Introduction

Clinicians in practice frequently overestimate the probability of bacterial pneumonia.^1^ Thirty to fifty of percent of patients initially suspected of having bacterial pneumonia either do not have pneumonia or have an alternative cause of symptoms.^2,3^ Misdiagnosis of bacterial infection almost universally leads to inappropriate antibiotic therapy, increasing the risk of adverse events.^4^

Pneumonia is a clinical diagnosis.^5^ No test is definitive, and clinician judgment is required to decide which tests to order and how to interpret the results. In this project, we sought to develop a diagnosis calculator to estimate numerical probability of community-acquired bacterial pneumonia based on objective clinical indicators and test results.

## Methods

The diagnosis calculator was built on a preexisting web interface (https://calculator.testingwisely.com) that can be customized with up to 3 incidence rates, 7 risk factors, and 5 diagnostic tests (up to 15 distinct inputs). Strong risk factors were assigned a likelihood ratio (LR) of 10, moderate risk factors LR 3, and minor risk factors LR 1.5.^6^ Factors that decrease likelihood of disease were assigned LR < 1. Diagnostic tests were characterized in terms of sensitivity and specificity.

Multiple open-ended searches of the literature were conducted by two infectious diseases physicians (RT, JDB) to identify US-based studies reporting values for required inputs. These searches were conducted using an integrative approach with the goal of synthesizing and summarizing data, rather systematically capturing all relevant studies.^7^ Examples of relevant keywords used to search included “diagnosis,” “pneumonia,” “bacterial,” “incidence,” “clinical factors,” “sensitivity,” and “specificity.” When a relevant study was found, its cited references were reviewed to identify additional studies. Values pulled from the literature were reviewed and adjusted by a local advisory panel with expertise in diagnostic stewardship (DJM, KCC), clinical epidemiology (ADH), and antimicrobial stewardship (EH, JB, KCC) to generate estimates used in the calculator. The pneumonia calculator was first published online on June 6, 2022.

## Results

### Incidence in specific populations

In the emergency department, 10–12% of adults with acute cough are diagnosed with pneumonia (see Supplemental Table 1 for references). With multiple vital sign abnormalities, incidence may be >60%. In outpatient clinics, about 6% of adults presenting with cough are diagnosed with pneumonia. Since about 1/3 of patients with microbiologically confirmed pneumonia have bacterial infection, we adjusted these incidence rates to 4% in the ED and 2% among outpatients (see Table 1 for incidence rates, likelihood ratios, and sensitivity and specificity of diagnostic tests).

**Table 1.** Influence of risk factors and diagnostic testing on diagnosis of bacterial pneumonia

Calculator Input	Estimate of Impact
Incidence	
1. In the ED with acute respiratory symptoms before chest X-ray	4%
2. Outpatients seen in clinic	2%
Risk factors (LR)	
1. Age ≥ 65	3
2. History of stroke or dementia	3
3. Immunocompromised	3
4. Other chronic comorbidity (i.e., COPD, CKD)	1.5
Syndrome (LR)	
1. Fever	3
2. Tachypnea	3
3. Hypoxemia (oxygen <94%)	3
4. Crackles or rales on chest exam	1.5
5. Noninfectious alternative cause of symptoms	0.5
Diagnostic test (clinical sensitivity/clinical specificity)	
1. Chest X-ray	65%/70%
2. CT chest	98%/75%
3. Sputum/lower respiratory culture^*^	30%/90%
4. Respiratory viral testing^**^	40%/52%
5. Procalcitonin 0.5 µg/L	55%/76%
6. Lung ultrasound	User dependent

CT, computed tomography. Immunocompromised was defined by active malignancy on chemotherapy, hematologic malignancy, or solid organ transplant.

* Depends on specimen type and quality, means of collection, and whether it is collected from a source that is expected to be contaminated, like an endotracheal tube that is known to be colonized.

** An indirect test for bacterial pneumonia. Bacterial/viral coinfection is possible.

### Risk factors

No strong risk factors were identified. Moderate risk factors include advanced age, history of stroke or dementia, immunocompromised status, fever, tachypnea, and hypoxemia. Minor risk factors included abnormalities on chest exam, which are subjective and may be difficult to reproduce, and other chronic comorbidity, which are nonspecific for bacterial infection. For instance, chronic obstructive pulmonary disease (COPD) has been associated with pneumonia but more commonly causes chronic bronchitis. Chronic kidney disease (CKD) has been associated with pneumonia but may also cause volume overload and pulmonary edema. Noninfectious cause of symptoms was associated with a likelihood ratio of less than 1.

### Imaging

Computed tomography (CT) imaging of the chest is commonly used to define pneumonia in the literature, and thus the false-negative rate was difficult to estimate. However, recognizing that chest CT may be falsely negative early in the course of illness, if a patient is dehydrated, or in the context of a low-quality exam, sensitivity was estimated to be 98%. Specificity of chest CT was informed by the proportion of results considered uncertain. Sensitivity and specificity of chest X-ray were determined based on an average of estimates across multiple studies of its accuracy in comparison to chest CT. Lung ultrasound can be accurate when performed by an experienced operator but may not be widely available.

### Microbiologic testing

Sensitivity and specificity of sputum culture are typically assessed for individual organisms, such as *Streptococcus pneumoniae*, rather than as a test for bacterial pneumonia. Thus, sensitivity of sputum culture was approximated by the diagnostic yield. Specificity was estimated based on the average of reported estimates for common pathogens. Specificity of lower respiratory culture is expected to be lower in the presence of an endotracheal tube or structural lung disease.

When performed, urinary antigen testing for *S. pneumoniae* and *Legionella pneumophila* is typically adjunctive to clinical culture. Urinary antigen testing is rarely studied or used as a standalone test. Though a positive result may increase clinical suspicion for bacterial pneumonia, a negative test does not influence decision making.

Respiratory viral panels using multiplex PCR were evaluated as an indirect test for bacterial pneumonia. Though coinfection is possible, a positive test for a viral pathogen decreases the likelihood of bacterial infection. When negative, viral infection remains possible. Sensitivity and specificity were inferred by computing likelihood ratios using the incidence of bacterial infection among patients with a positive viral test, a negative test, and no test performed.

### Serum procalcitonin

The sensitivity and specificity of serum procalcitonin to differentiate bacterial from viral pneumonia were reported in a systematic review and meta-analysis. The most common threshold value is 0.5 µg/L, though other thresholds have been used. Procalcitonin can guide initiation and duration of antibiotic therapy, suggesting it distinguishes bacterial pneumonia from both viral infection and noninfectious conditions.

## Discussion

We developed a diagnosis calculator to explore how clinical information impacts the pre- and post-test probability of bacterial pneumonia. Though this tool can be used to calibrate diagnostic decision making, inform test ordering, support interpretation of test results, and provide medical education (https://calculator.testingwisely.com), it should be considered preliminary pending further validation. The estimated probabilities it produces require adjustment based on subjective input from the clinician-user.

Clinicians in practice routinely misestimate the probability of bacterial infection.^8^ The medical literature contributes to this problem by presenting data in a format that is not relevant or applicable to clinical decision making. The diagnosis calculator bridges this gap by converting data pulled from the medical literature into clinically relevant risk estimates. However, clinician intuition is still required. Clinical decision making tends to be safer when structured models using objective data are combined with clinician gestalt.

Diagnostic errors occur because of both bias and noise in decision making. Bias is introduced by clinicians’ tendency to overestimate the probability of disease. Noise comes from variation in training backgrounds, clinical experiences, attitudes, and beliefs that influence both likelihood of ordering tests and interpretation of the results.^9,10^ The diagnosis calculator can reduce both bias and noise by providing an accurate and consistent anchor for initial probability estimates.

There is no gold standard test for bacterial pneumonia. Chest CT is frequently used as a reference but cannot reliably differentiate between viral and bacterial infection.^3,11^ Culture can confirm a microbiologic diagnosis but has limited sensitivity on sputum.^12^ In many cases, it may never be completely clear whether a patient had bacterial pneumonia or not. This lack of certainty likely contributes to overuse of antibiotics for pneumonia and may delay the diagnosis of noninfectious conditions.

The main limitation of this project is that we did not conduct a systematic review or meta-analysis. Instead, we defined our inputs as a clinician would: by conducting an open-ended search and discussing the findings with peers and trusted experts. Thus, we were unable to generate precise estimates of LRs with confidence intervals. Instead, risk factors were organized into tiers and assigned the associated LR. Actual rates, LRs, and test characteristics may vary by severity or context. For instance, the sensitivity of testing may increase when multiple specimens are collected. Finally, though our diagnosis calculator informs an objective estimate of the probability of bacterial pneumonia, thresholds for treatment with antibiotics remain subjective. The decision to prescribe antibiotics is complicated and frequently multifactorial.

## Conclusion

We compiled clinical data from the medical literature into a calculator to support the diagnosis of community-acquired bacterial pneumonia. Though preliminary, this calculator can be used by clinicians to calibrate diagnostic decision making and for educational purposes.

## Supplementary material

For supplementary material accompanying this paper visit http://doi.org/10.1017/ash.2023.408. click here to view supplementary material
