# Supplementary material for: Developing a diagnosis calculator to estimate the probability of bacterial pneumonia
Source: Antimicrob Steward Healthc Epidemiol. 2023 Aug 7;3(1):e137. doi: 10.1017/ash.2023.408 (PMC10428145; doi:10.1017/ash.2023.408)
Supplement: Supplementary file 1 [file S2732494X23004084sup001.docx]

**Supplemental Table 1.** Studies Contributing Calculator Inputs

|  | **Relevant Studies** |
| --- | --- |
| Incidence | 1. Flanders SA, Stein J, Shochat G, et al. Performance of a bedside C-reactive protein test in the diagnosis of community-acquired pneumonia in adults with acute cough. *Am J Med.* 2004;116(8):529-535. 2. Emerman CL, Dawson N, Speroff T, et al. Comparison of physician judgment and decision aids for ordering chest radiographs for pneumonia in outpatients. *Ann Emerg Med*. 1991;20(11):1215-1219. 3. Jain S, Self WH, Wunderink RG, et al. Community-Acquired Pneumonia Requiring Hospitalization among U.S. Adults. *N Engl J Med.* 2015;373(5):415-427. |
| Risk factors | 1. Nolt BR, Gonzales R, Maselli J, Aagaard E, Camargo CA, Jr., Metlay JP. Vital-sign abnormalities as predictors of pneumonia in adults with acute cough illness. *Am J Emerg Med.* 2007;25(6):631-636. 2. Huijskens EGW, Koopmans M, Palmen FMH, van Erkel AJM, Mulder PGH, Rossen JWA. The value of signs and symptoms in differentiating between bacterial, viral and mixed aetiology in patients with community-acquired pneumonia. *J Med Microbiol.* 2014;63(Pt 3):441-452. 3. Kishore AK, Vail A, Chamorro A, et al. How is pneumonia diagnosed in clinical stroke research? A systematic review and meta-analysis. *Stroke.* 2015;46(5):1202-1209. 4. Mullerova H, Chigbo C, Hagan GW, et al. The natural history of community-acquired pneumonia in COPD patients: a population database analysis. *Respir Med.* 2012;106(8):1124-1133. 5. Chou CY, Wang SM, Liang CC, et al. Risk of pneumonia among patients with chronic kidney disease in outpatient and inpatient settings: a nationwide population-based study. *Medicine (Baltimore).* 2014;93(27):e174. |
| Diagnostic Testing | 1. Syrjala H, Broas M, Suramo I, Ojala A, Lahde S. High-resolution computed tomography for the diagnosis of community-acquired pneumonia. *Clin Infect Dis.* 1998;27(2):358-363. 2. Hayden GE, Wrenn KW. Chest radiograph vs. computed tomography scan in the evaluation for pneumonia. *J Emerg Med.* 2009;36(3):266-270. 3. Claessens YE, Debray MP, Tubach F, et al. Early Chest Computed Tomography Scan to Assist Diagnosis and Guide Treatment Decision for Suspected Community-acquired Pneumonia. *Am J Respir Crit Care Med.* 2015;192(8):974-982. 4. Prendki V, Scheffler M, Huttner B, et al. Low-dose computed tomography for the diagnosis of pneumonia in elderly patients: a prospective, interventional cohort study. *Eur Respir J.* 2018;51(5):1702375. 5. Makhnevich A, Sinvani L, Cohen SL, et al. The Clinical Utility of Chest Radiography for Identifying Pneumonia: Accounting for Diagnostic Uncertainty in Radiology Reports. *AJR Am J Roentgenol.* 2019;213(6):1207-1212. 6. Garin N, Marti C, Scheffler M, Stirnemann J, Prendki V. Computed tomography scan contribution to the diagnosis of community-acquired pneumonia. *Curr Opin Pulm Med.* 2019;25(3):242-248. 7. Self WH, Courtney DM, McNaughton CD, Wunderink RG, Kline JA. High discordance of chest x-ray and computed tomography for detection of pulmonary opacities in ED patients: implications for diagnosing pneumonia. *Am J Emerg Med.* 2013;31(2):401-405. 8. Fukuyama H, Yamashiro S, Kinjo K, Tamaki H, Kishaba T. Validation of sputum Gram stain for treatment of community-acquired pneumonia and healthcare-associated pneumonia: a prospective observational study. *BMC Infect Dis.* 2014;14:534. 9. Ogawa H, Kitsios GD, Iwata M, Terasawa T. Sputum Gram Stain for Bacterial Pathogen Diagnosis in Community-acquired Pneumonia: A Systematic Review and Bayesian Meta-analysis of Diagnostic Accuracy and Yield. *Clin Infect Dis.* 2020;71(3):499-513. 10. Del Rio-Pertuz G, Gutierrez JF, Triana AJ, et al. Usefulness of sputum gram stain for etiologic diagnosis in community-acquired pneumonia: a systematic review and meta-analysis. *BMC Infect Dis.* 2019;19(1):403. 11. García-Vázquez E, Marcos MA, Mensa J, et al. Assessment of the Usefulness of Sputum Culture for Diagnosis of Community-Acquired Pneumonia Using the PORT Predictive Scoring System. *Arch Intern Med.* 2004;164(16):1807-1811. 12. Del Rio-Pertuz G, Gutiérrez JF, Triana AJ, Molinares JL, Robledo-Solano AB, Meza JL, Ariza-Bolívar OM, Acosta-Reyes J, Garavito A, Viasus D, Carratalà J. Usefulness of sputum gram stain for etiologic diagnosis in community-acquired pneumonia: a systematic review and meta-analysis. *BMC Infect Dis*. 2019 May 10;19(1):403. doi: 10.1186/s12879-019-4048-6. 13. Jain S, Self WH, Wunderink RG, et al. Community-Acquired Pneumonia Requiring Hospitalization among U.S. Adults. *N Engl J Med.* 2015;373(5):415-427. 14. Dionne M, Hatchette T, Forward K. Clinical utility of a Legionella pneumophila urinary antigen test in a large university teaching hospital. *Can J Infect Dis.* 2003;14(2):85-88. 15. West DM, McCauley LM, Sorensen JS, Jephson AR, Dean NC. Pneumococcal urinary antigen test use in diagnosis and treatment of pneumonia in seven Utah hospitals. *ERJ Open Res.* 2016;2(4). 16. Klompas M, Imrey PB, Yu PC, et al. Respiratory viral testing and antibacterial treatment in patients hospitalized with community-acquired pneumonia. *Infect Control Hosp Epidemiol.* 2021;42(7):817-825. 17. Kamat IS, Ramachandran V, Eswaran H, Guffey D, Musher DM. Procalcitonin to Distinguish Viral From Bacterial Pneumonia: A Systematic Review and Meta-analysis. *Clin Infect Dis.* 2020;70(3):538-542. 18. Rhee C. Using Procalcitonin to Guide Antibiotic Therapy. *Open Forum Infect Dis.* 2016;4(1). 19. Schuetz P, Wirz Y, Sager R, et al. Procalcitonin to initiate or discontinue antibiotics in acute respiratory tract infections. *Cochrane Database Syst Rev.* 2017;10(10):Cd007498. 20. Chavez MA, Shams N, Ellington LE, Naithani N, Gilman RH, Steinhoff MC, Santosham M, Black RE, Price C, Gross M, Checkley W. Lung ultrasound for the diagnosis of pneumonia in adults: a systematic review and meta-analysis. *Respir Res.* 2014 Apr 23;15(1):50. doi: 10.1186/1465-9921-15-50. 21. Reissig A, Copetti R, Mathis G, et al. Lung ultrasound in the diagnosis and follow-up of community-acquired pneumonia: a prospective, multicenter, diagnostic accuracy study. *Chest*. 2012;142(4):965-972. |
